# Head and Neck Cancer: United Kingdom National Multidisciplinary Guidelines, Sixth Edition – CORRIGENDUM

**DOI:** 10.1017/S0022215125102491

**Published:** 2025-07

**Authors:** Jarrod J Homer, Stuart C Winter

Upon publication the affiliation NIHR UCLH Biomedical Research Centre was not listed for author Stefano Fedele. The full affiliation list for Stefano Fedele is:

1) UCL Eastman Dental Institute, University College London, London, UK

2) NIHR UCLH Biomedical Research Centre, London, UK

The article has been updated

## References

[ref1] Homer JJ, Winter SC. et al Head and Neck Cancer. United Kingdom National Multidisciplinary Guidelines, Sixth Edition. The Journal of Laryngology & Otology. 2024;138(S1):S1–S224. doi:10.1017/S0022215123001615 38482835

